# Knowledge, Attitude, Perception and Current Practices of Health Personnel in Managing Post-Stroke Delirium in a New Stroke Centre in Malaysia

**DOI:** 10.21315/mjms2023.30.4.14

**Published:** 2023-08-24

**Authors:** Hanin Nabila Mohd Yosli, Wei Hong, Khairunnisa Kazura, Noor Hafizah Abdul Salim, Ahmad Luqman Md Pauzi, Hazwan Mat Din, Hakimah Sallehuddin, Zahira Zohari, Halimatus Sakdiah Minhat

**Affiliations:** 1 Faculty of Medicine and Health Sciences, Universiti Putra Malaysia, Selangor, Malaysia; 2 Department of Emergency, Hospital Sultan Abdul Aziz Shah, Universiti Putra Malaysia, Selangor, Malaysia; 3 Malaysian Research Institute on Ageing (MyAgeing™), Universiti Putra Malaysia, Selangor, Malaysia; 4 Geriatric Unit, Department of Medicine, Faculty of Medicine and Health Sciences, Universiti Putra Malaysia, Selangor, Malaysia; 5 Department of Community Health, Faculty of Medicine and Health Sciences, Universiti Putra Malaysia, Selangor, Malaysia

**Keywords:** delirium, knowledge, health personnel, acute stroke, current practice

## Abstract

**Introduction:**

Despite the high prevalence of post-stroke delirium in Malaysia, there are no studies on good practices related to its management. This study aimed to analyse the knowledge, attitude, perception, and factors associated with current practices related to delirium in acute stroke patients among health personnel at Hospital Sultan Abdul Aziz Shah (HSAAS) (formerly known as Hospital Pengajar Universiti Putra Malaysia).

**Methods:**

This cross-sectional study was conducted from 26 April 2021 to 9 May 2021 (17 weeks). All health personnel from various departments managing patients with acute stroke in our centre were invited to participate. An online questionnaire was disseminated to assess their knowledge, attitude, perception, and current practices concerning delirium. Multiple logistic regression was used to examine the association between the independent and dependent variables. The level of significance was set at *P* < 0.05.

**Results:**

The response rate was 22.49% (47 of 209 participants). More than half (61.7%, *n* = 29) had good current practices towards delirium in acute stroke patients. A significant association was found between knowledge and current practices related to delirium (*P* = 0.024). However, there was no significant association between current practices and sociodemographic factors (age, ethnicity, gender and job position), attitude, perceptions, screening barriers, or health service organisation.

**Conclusion:**

Most respondents had good current practices and knowledge in managing post-stroke delirium. Therefore, upskilling health personnel for managing this illness is essential to ensure good post-stroke care and improve prognosis related to delirium.

## Introduction

Delirium is a potentially reversible organic brain syndrome. Elfeky and Ali (1) stated that delirium is prevalent across different treatment settings and is more frequent in critically ill patients, the elderly and patients with cognitive impairment. The prevalence of delirium among patients aged ≥ 65 years old admitted to medical wards at a large teaching hospital in Malaysia was 26.4%, increasing to 56% in those aged ≥ 85 years old (2).

Establishing a stroke care unit through the Regional Emergency Stroke Quick-Response (RESQ) network strategy is an effort to reduce the difficulties associated with acute stroke management. Hospital Sultan Abdul Aziz Shah (HSAAS) is a tertiary centre with a primary RESQ unit. The management involves a multidisciplinary team, ranging from critical management to rehabilitation (3). These patients are potentially managed by health personnel from the Medical and Neurology Department; Anaesthesiology and Critical Care; RESQ; Otolaryngology, Head and Neck (ENT) Department (speech therapy); Dietetics Department, Psychiatry Department and Rehabilitation Medicine Department.

Delirium may affect 10%–30% of patients in the acute phase of stroke. It is usually associated with higher mortality, extended hospitalisation and dependency after discharge (4). It may predict long-term cognitive impairment in survivors of critical conditions and is associated with cognitive decline over 1–3 years after hospital discharge (5). The signs and symptoms of delirium may begin as early as a few hours and may last for days and months. Symptoms may fluctuate throughout the day and patients can sometimes revert to their normal selves. The primary signs and symptoms include reduced awareness of the environment, poor thinking skills, behavioural changes and emotional disturbances.

Based on the Malaysian Clinical Practice Guideline (CPG) Management of Acute Ischaemic Stroke 2020 (6), it is recommended that all post-stroke patients be screened for delirium throughout hospitalisation. In handling delirium, good current practices are essential, involving all appropriate considerations and procedures, including early preventative management, sufficient staff and screening tools, good perception of practitioners, involvement of a multidisciplinary team, incorporation of delirium as a primary training curriculum for health personnel and regular assessment for delirium. These are crucial factors that contribute to good practices (7).

A study has shown that up to two-thirds of nurses caring for intensive care unit (ICU) patients do not recognise delirium (1). Health personnel face significant problems because of the absence of assessment tools and subsequently, delirium is misdiagnosed, treated inappropriately or even neglected (8). Therefore, there is a need to assess the current practices concerning delirium, among health personnel, to understand the gaps in management and identify future needs, such as training workshops to improve the quality of care. To date, research on current practices and the management of post-stroke delirium, among health personnel, is scarce, and to the best of our knowledge, this is the first study performed in Malaysia. This study aimed to determine the factors associated with current practices related to delirium in acute stroke patients, among health personnel at HSAAS.

## Methods

This cross-sectional study was conducted using an online questionnaire generated using Google Forms (Appendix). Data were collected from 26 April 2021 to 9 May 2021 (17 weeks). A total of 209 participants were invited via emails from the hospital website, involving the following departments in the HSAAS at the Universiti Putra Malaysia: Medical and Neurology; Radiology; RESQ; Rehabilitation Medicine; Dietetics; Anaesthesiology and Critical Care; ENT and Psychiatry. The departments were chosen based on the potential involvement of their health personnel in managing post-stroke delirium at some point in daily clinical practice. The response rate was 22.49% (47 of 209 invited participants).

Ethical clearance was obtained from the Ethics Committee of Universiti Putra Malaysia for this study involving human subjects. Respondents were asked to provide written informed consent to participate in this study and were made aware that the information obtained from the questionnaire was confidential.

### Sample Size Calculation

The sample size of this study was calculated using the one-proportion formula for prevalence study:


$$n=(Z^{2}\times \text{P}\times (1-\text{P}))/{\text{e}}^{2}$$(
9)

The minimum required sample size was 128 respondents (*Z* = 1.96 at 0.05 significance level, P = proportion of 90.84% good practices among health personnel, related to delirium, *e* = precision at 5%) (3). However, due to coronavirus disease (COVID-19) and time constraints, only 47 respondents were recruited. This sample size yielded approximately 8% data precision, which is considered acceptable (10).

### Online Questionnaire

The questionnaire was written in English. It consisted of seven sections: i) sociodemographic background, ii) knowledge of delirium, iii) current practices, iv) perceptions, v) attitude, vi) screening barriers, and vii) health service organisations. They were adapted from published studies by Xing et al. (9), Monfared et al. (10), Herrero et al. (11) and, Elfeky and Ali (1). The scoring method was adapted from that described by Monfared et al. (10). The questionnaire content was evaluated using face validation and content validity. The reliability of this study was also evaluated: i) content validity was assessed by two experts in the care of older adults (a geriatrician and community health specialist), ii) face validity was assessed through the distribution of the questionnaire draft to six health personnel in selected departments at the HSAAS to assess the quality of the questionnaire components. All comments were incorporated into the revised version.

### Operational Definitions

The departments were divided into two groups: i) Medical/Neurology/RESQ and ii) another group collectively consisting of Radiology, Rehabilitation, Dietetics, Anaesthesiology, ENT and Psychiatry. The former group handles the care of stroke patients during pre-admission and in-hospital admission.

The job positions were also divided into specialists, consultants, medical officers, and supporting health staff, consisting of nurses, physiotherapists, occupational therapists, dieticians and speech therapists. This further division denotes the level of education and tasks.

#### Current Practices

Current practices toward delirium are classified into good and poor practices. It was considered good practice when the respondent correctly answered three or more questions (maximum score = 4). The questions were regarding their opinions on appropriate considerations and approaches to handling delirium in stroke patients.

Good current practices involve all appropriate considerations and practices in handling delirium in stroke patients, including early preventative management, sufficient staff and screening tools, the perceptions of the practitioners and involvement of a multidisciplinary team. Adding delirium as a primary training curriculum for health personnel and regular assessment for delirium are some crucial factors contributing to good practices (Malaysian Society of Geriatric Medicine Position: Delirium, 2017) (7).

#### Knowledge Regarding Delirium

Knowledge is defined as the ability to assess delirium using standard methods. If most of the answers provided by the respondents were correct or if they had received any prior training on delirium, they were considered to have good knowledge of delirium.

#### Perceptions

Perception is defined as the way in which health personnel recognise and interpret the symptoms of delirium with appropriate and suitable conditions and diagnoses. The perceptions were then characterised as strongly disagree, disagree, neither/nor agree, agree and strongly agree. Good perception was defined as a score of ≥ 13.

#### Attitude

Attitude is the health personnel’s reaction towards acute stroke patients with delirium, influenced by their view of selected issues. It was divided into yes, no, and uncertain. Good attitude was defined as a score of ≥ 7.

#### Screening Barriers

Barriers that prevent health personnel from screening delirium in acute stroke patients are based on knowledge, heavy workload, lack of screening tools, lack of evaluation, lack of cooperation and time consumption among others (14). Responses were yes, no or not sure. Screening barriers were categorised into fewer screening barriers (1–6 score) or several screening barriers (7–12 score).

#### Health Service Organisation

The health service organisation is defined as the degree of capability and environment of the hospital setting, contributing to practices related to delirium (11). If both the questions were answered as ‘yes’, it was considered a good health service organisation for managing delirium.

## Statistical Analysis

Descriptive statistics are presented as frequencies and percentages for sociodemographic data. Simple logistic regression was conducted before multivariate analysis. Variables with *P* < 0.25 in univariate analysis and considered clinically or biologically significant by the researchers, were entered into multiple logistic regression models and treated as confounders (if they were not significant factors). Multiple logistic regression with the enter method was used to examine the association between independent and dependent variables, which enabled all input variables to be entered simultaneously in one block of the model. In this study, researchers intended to control all potential confounders; although they were not statistically significant at the univariate level, they were clinically or biologically significant (12). Regression analysis demonstrated the odds ratio, 95% confidence interval (CI) and *P*-value. The level of significance was set at *P* < 0.05. The Homer-Lemeshow test of goodness-of-fit, classification table and receiver operating characteristic curve analysis were performed for the assumption of multiple logistic regression. Data analysis was performed using the Statistical Package for the Social Sciences (SPSS) version 26.0.

## Results

### Demographics

Of the 209 invited participants, 47 (22.5%) responded. Many respondents were from medical, neurology and RESQ departments (57.4%, *n* = 27) (Table 1). The highest number of respondents were allied health staff (46.8%), followed by specialists/consultants (31.9%) and medical officers (21.3%).

Twenty-eight respondents (59.6%) were > 31 years old. Most of them were Malays (80.9%, *n* = 38), followed by Indians and Chinese (12.8% and 6.4%, respectively). More than two-thirds of the respondents were female (70.2%, *n* = 33). Table 2 compares knowledge, perception, attitude, current practices, and health service organisation between the level of position of health personnel.

Based on their clinical experience, 27 health personnel (57.4%, *n* = 27) had < 10 years of clinical experience. In comparison, 18 (38.3%) had 11–20 years of experience and the remaining (4.3%) had ≥ 21 years of experience in dealing with acute stroke patients with delirium.

### Knowledge Regarding Delirium

Sixteen (34%) health personnel had good knowledge of delirium, while 31 (66%) had poor knowledge. Specialists and consultants had good knowledge, while many medical officers and supporting health staff had insufficient knowledge (Table 2).

All the participants were aware of the risk factors of dementia, including a history of dementia, older age and stroke. Only 15 health personnel (31.9%) received training for delirium. However, almost half of them (48.9%, *n* = 23) knew when and how to screen for delirium. Many participants (81.9%) were unaware of the prevalence of delirium among stroke patients in an acute stroke unit.

### Perceptions

The data indicated that 44 of the 47 health personnel (93.6%) had a good perception of delirium, and 3 others (6.4%) had a poor perception of delirium in acute stroke patients. Approximately half the health personnel (48.9%, *n* = 23) agreed that delirium is common in patients with acute stroke. Twenty-three people (43.8%) perceived delirium as an under-diagnosed problem and 18 (38.8%) indicated that delirium was challenging to assess in patients with stroke. The opinion was divided when asked about their perception regarding the association between delirium and high mortality, with about one-third of the participants (34%, *n* = 16) agreeing to some extent. Only one participant (2.1%) strongly agreed that delirium rarely presents as agitation.

### Attitude

A high percentage (97.9%, *n* = 46) of the participating health personnel had a good attitude regarding post-stroke delirium patients, except for one with a poor attitude (2.1%). They did not think screening for delirium was a waste of time and 91.5% (*n* = 43) believed that screening should be performed routinely. Nearly half of the participants (46.8%, *n* = 22) perceived delirium as a normal sequela of an acute stroke. However, 20 health personal (42.6%) perceived the screening tools as complicated.

### Current Practices

Based on current practices related to delirium in acute stroke patients, 29 (61.7%) respondents had good practices related to delirium. The best practice was among supporting health officers (41.4%, *n* = 12), followed by specialists and consultants (37.9%, *n* = 11). Based on the Table 2, the participants mostly agreed on appropriate considerations. Current practices in the management of delirium include early detection through a standardised screening tool for high-risk patients (93.6%, *n* = 44). Delirium care should be included as one of the basic training curricula for health personnel (70.2%, *n* = 33) and a proper structured evaluation procedure is required according to the hospital policy for delirium (61.7%, *n* = 29). More than half of the health personnel (59.6%, *n* = 28) concurred that adequate health personnel in managing stroke patients is important to avoid under-diagnosed delirium in acute stroke patients.

### Screening Barriers

Thirty-two (68.1%) participants thought that there were many screening barriers related to post-stroke delirium. The data show that the highest number of supporting health staff thought many screening barriers exist (34%, *n* = 16). The majority admitted that one of the barriers was insufficient knowledge and training regarding delirium in acute stroke patients (80.1%, *n* = 38).

### Health Service Organisation

The survey also showed that a few respondents acknowledged the hospital’s policies and guidelines for preventing and managing delirium (19.1%, *n* = 9). Only 14 (29.8%) respondents knew about the guidelines in HSAAS for delirium risk identification, prevention and management. Approximately half agreed that the equipment and devices (low-rise beds, call bells and clocks) are sufficient.

### Factors Associated with Current Practice

The associations between current practices related to post-stroke delirium, among HSAAS health personnel, are shown in Table 3. This study showed a significant association between the respondents’ knowledge and current practices (*P* = 0.024).

Otherwise, there was no significant association between socio-demographic background, clinical experience, perception, attitude, screening barriers, and health service organisation and current practices.

Table 4 summarises the questions for each section and the responses.

## Discussion

Delirium in acute stroke remains under-recognised, under-diagnosed, and under-treated despite advancements in the diagnosis and management of stroke. The most important finding of this study was the significant association between knowledge and current practices related to delirium in patients with acute stroke, among health personnel in HSAAS. Good knowledge can lead to a better understanding of delirium, as shown by Monfared et al. (10). Knowledge deficits among medical doctors and supporting staff were detected in this study. Hence, it is essential to strengthen it with more training and teaching. Adequate knowledge and accurate understanding can encourage a medical practitioner to be more alert in detecting patients with delirium to avoid under-diagnosing delirium, especially in hypoactive delirium patients. This could help them deal with such patients in a better manner.

Considerable knowledge among specialists and consultants may be related to their level of education and work experience. Monfared et al. (10) also stated that age and clinical work experience are significantly associated with nurses’ knowledge about delirium, which increases with age and work experience. However, our study showed no association between socio-demographic factors such as age, job position and current practice related to delirium in acute stroke patients, among HSAAS health personnel. The difference in results may be due to the low number of respondents, and the age of the health personnel is not equally distributed, with almost half of our respondents (46%) in the age group of 31 years old–40 years old.

Many participants sensed the difficulties and barriers in screening for delirium. Most believe that there is no structured evaluation procedure and a lack of evaluation tools. This issue was also addressed by Elliot et al. (13), where even though multiple screening tools for delirium are available (the 4 ‘A’s test [4-AT] and Confusion Assessment Method [CAM)-ICU] (14, 15), they are still not widely used. Again, the main reason is insufficient knowledge and training.

When the perception of managing delirium improves, current practices toward delirium will also improve. In our study, we found no significant association between perception and current practices related to delirium in acute stroke patients, among health personnel in HSAAS. Hickin et al. (16) found an association between perception of delirium and current practices. Elfeky and Ali (1) also found that health personnel have a good perception of delirium, which correlates with their current practices. They discovered that delirium is under-diagnosed in the ICU and requires active intervention. However, our findings contradict those of previous studies. These different results may be due to the small number of participants in our study and the results may not be generalisable.

However, there is a lack of research on attitude and current practices related to delirium. Our study results showed no association between attitude and current practices related to delirium in acute stroke patients, among health personnel in HSAAS. However, a survey by Monfared et al. (10) reported an association between attitude and current practices related to delirium in acute stroke patients, among health personnel. It should be noted that a positive attitude will improve the current practices towards patients. However, our findings contradict those of previous studies. This may be due to the different cultures of our target population in prior research, which results in having a variety of viewpoints on an individual or an item, from positive to negative, or from loving to detesting.

Another concern is the low number of health service organisations related to delirium in acute stroke patients in HSAAS. According to the Australian Commission on Safety and Quality in Health Care Delirium 2018 (17), the best practice for preventing delirium is through a health service organisation that targets at-risk groups of delirium patients. This is because it has systems for risk recognition, prevention, and management of delirium, which help medical practitioners to detect delirium efficiently and prevent further complications in delirium patients. In contrast, our research found no significant association comparable to the basis of health service organisations. This might be due to the recent establishment of HSAAS, which makes health personnel unaware of the guidelines regarding delirium in HSAAS or possibly because of the experience of health personnel, as most of our respondents have a clinical experience of < 10 years. Therefore, it is imperative to implement further training and continuous medical education.

## Conclusion

We found that most of the health personnel who responded to the survey had good current practices, perceptions, and knowledge regarding delirium in patients with acute stroke. There was also a significant association between knowledge and current practices related to delirium in acute stroke patients, among health personnel.

This allowed us to identify and implement the need for a training module on good practices related to delirium, for health personnel working directly or indirectly with patients admitted for acute stroke. Further studies should be conducted to evaluate the knowledge regarding current practices among health personnel.

Research on suitable delirium assessment tools and cognitive screening in Malaysian emergency settings for patients admitted with acute stroke is recommended.

## Strength and Limitations

The main limitations of this study are its small sample size and limited research time. The low response rate was because the questionnaire targeted only health personnel managing stroke patients. They are related to critical departments involved in frequent monitoring of emergency patients, which limited their available time to answer the questionnaire. Furthermore, during the pandemic, most health personnel were busy managing the high number of COVID-19 admissions, and some were further deployed to another centre.

Following the policy of maintaining social distancing, the questionnaire could only be distributed online instead of physically. Self-reporting responses may have inaccuracies due to response bias, which could be caused by a lack of recollection of clinical experience or misunderstanding of the questions when answering online. Potential bias also exists because of sample heterogeneity.

In addition, our study was limited to the departments in HSAAS, which may affect the generalisability of the study. The strength of this study is that it is the first in Malaysia to investigate factors associated with current practices in post-stroke delirium in a stroke centre.

## Figures and Tables

**Table 1:** Frequency of respondents for each factor

Variables	Frequency (*n*)	%
Gender		
Male	14	29.8
Female	33	70.2
Age group (years old)		
30 and below	19	40.4
31 and above	28	59.6
Ethnicity		
Malay	38	80.9
Chinese	3	6.4
Indian	6	12.8
Position		
Specialist/Consultant	15	31.9
Medical doctor	10	21.3
Health staff	22	46.8
Department		
Medical/Neurology/RESQ	27	57.4
Others	20	42.6
Knowledge		
Good	16	34.0
Poor	31	66.0
Perception		
Good	44	93.6
Poor	3	6.4
Attitude		
Good	46	97.9
Poor	1	2.1
Clinical experience		
Less than 10 years	27	57.4
11–20 years	18	38.3
20–30 years	2	4.3
Screening barriers related to delirium		
Little screening barrier	15	31.9
Many screenings barrier	32	68.1
Health service organisation of delirium		
Yes and Yes	9	19.1
Yes and No/No and No	38	80.9
Current practices		
Good	29	61.7
Poor	18	38.3

Note: RESQ = regional emergency stroke quick response

**Table 2:** Distribution of study variables among the position and departments of the respondents

Variable	Position, *n* (%)			Department, *n* (%)	
	
	Specialist/Consultant	Medical doctor	Supporting staff	Medical/Neuro/RESQ	Others
Knowledge					
Good	11 (73.3)	1 (10)	4 (18.2)	11 (40.7)	5 (25.0)
Poor	4 (26.7)	9 (90.0)	18 (81.8)	16 (59.3)	15 (75.0)
Perception					
Good	15 (100)	9 (90.0)	20 (90.9)	25 (92.6)	19 (95.0)
Poor	0 (0.0)	1 (10.0)	2 (9.1)	2 (7.40	1 (5.0)
Attitude					
Good	15 (100.0)	10 (100.0)	21 (95.5)	27 (100.0)	19 (95.0)
Poor	0 (0.0)	0 (0.0)	1 (4.5)	0 (0.0)	1 (5.0)
Clinical experience					
Less than 10 years	1 (6.7)	9 (90.0)	17 (77.3)	12 (44.4)	15 (75.0)
11–20 years	12 (80.0)	1 (10.0)	5 (22.7)	14 (51.9)	4 (20.0)
20–30 years	2 (13.3)	0 (0.0)	0 (0.0)	1 (3.7)	1 (5.0)
Screening barriers related to delirium					
Little	7 (46.7)	2 (20.0)	6 (27.3)	9 (33.3)	6 (30.0)
Many	8 (53.3)	8 (80.0)	16 (72.7)	18 (66.7)	14 (70.0)
Health service organisation of delirium					
Yes and Yes	3 (20.0)	2 (20.0)	4 (18.2)	8 (29.6)	1 (5.0)
Yes and No/No and No	12 (80.0)	8 (80.0)	18 (81.8)	19 (70.4)	19 (95.0)
Current practices					
Good	11 (73.3)	6 (60.0)	12 (54.5)	17 (63.0)	12 (60.0)
Poor	4 (26.7)	4 (40.0)	10 (45.5)	10 (37.0)	8 (40.0)

Note: RESQ = regional emergency stroke quick response

**Table 3:** Association between current practices related to post-stroke delirium patients and study variables among health personnel

Variable	Current practices, *n* (%)		SLR^a^		MLR^b^	
		
	Good	Poor	OR (95% CI)	*P*-value	OR (95% CI)	*P*-value
Gender						
Male	9 (64.3)	5 (35.7)	Reference level		Reference level	
Female	21 (60.6)	12 (39.4)	0.86 (0.23, 3.13)	0.812	2.19 (0.36, 13.49)	0.397
Age group (years old)						
29 and below	10 (52.9)	9 (47.4)	Reference level		Reference level	
30 and above	19 (67.9)	9 (32.1)	0.52 (0.16, 1.75)	0.294	0.27 (0.02, 3.04)	0.288
Ethnicity						
Malay (Ref)	24 (63.2)	14 (36.8)	Reference level		Reference level	
Non-Malay	5 (55.6)	4 (44.4)	1.37 (0.32, 5.97)	0.674	1.21 (0.15, 9.860)	0.860
Position						
Specialist/Consultant	11 (73.3)	4 (26.7)	Reference level		Reference level	
Medical doctor	6 (60.0)	4 (40.0)	0.55 (0.10, 3.00)	0.486	1.75 (0.08, 37.81)	0.723
Health staff	12 (54.5)	10 (45.5)	0.44 (0.11, 1.80)	0.252	0.75 (0.05, 11.79)	0.836
Department						
Medical/Neurology/RESQ	17 (63.0)	10 (37.0)	Reference level		Reference level	
Others	12 (60.0)	8 (40.0)	1.13 (0.35, 3.72)	0.836	2.01 (0.36, 11.060)	0.424
Knowledge						
Good knowledge	14 (87.5)	2 (12.5)	Reference level		Reference level	
Poor knowledge	15 (48.4)	16 (51.6)	0.13 (0.03, 0.69)	0.016*	0.06 (0.01, 0.69)	0.024*
Perception						
Good perception	27 (61.4)	17 (38.6)	Reference level		Reference level	
Poor perception	2 (66.7)	1 (33.3)	1.26 (0.11, 14.98)	0.855	2.46 (0.03, 185.380)	0.684
Attitude						
Good attitude	28 (60.9)	18 (39.1)	Not applicable		Not applicable	
Poor attitude	1 (100.0)	0 (0.0)				
Clinical experience						
Less than 10 years	14 (51.9)	13 (48.1)	Reference level		Reference level	
11–20 years	14 (77.8)	4 (22.2)	3.25 (0.85. 12.45)	0.085	7.02 (0.44, 112.70)	0.169
20–30 years	1 (50.0)	1 (50.0)	0.93 (0.05, 16.42)	0.960	0.31 (0.003, 38.030)	0.117
Screening barriers related to delirium						
Little screening barrier	7 (46.7)	8 (53.3)	Reference level		Reference level	
Many screening barriers	22 (68.8)	10 (31.2)	2.51 (0.71, 8.86)	0.152	4.37 (0.69, 27.72)	0.117
Health service organisation						
Yes and Yes	6 (66.7)	3 (33.3)	Reference level		Reference level	
Yes and No/No and No	24 (60.5)	14 (39.5)	0.77 (0.17, 3.54)	0.734	0.51 (0.04, 7.070)	0.614

Notes:

a Simple logistics regression;

b Multiple logistics regression (Enter method); Dependent variable coding: Good current practice = 1, Poor current practice = 0; Hosmer-Lemeshow test, *P* = 0.880; Classification table, 78.7%; Nigelkerke R^2^, 42.0%; *n* = frequency; OR = odds ratio; 95% CI = 95% confidence interval;

* indicates a statistically significant association between knowledge and current practices with *P* < 0.05 in both SLR and MLR, suggesting that the level of knowledge significantly influences the current practices regardless of the complexity of the regression model used

**Table 4:** Descriptive statistics of study variable questions and the responses

Variable	*Yes *n* (%)
Knowledge on delirium	
Have you received any training in delirium?	15 (31.9)
Among risk factors of delirium include a history of dementia, older age and stroke	47 (100.0)
I know how and when to do screening for delirium	23 (48.9)
There is low prevalence of delirium among stroke patients in acute stroke units	9 (19.1)
Perception on delirium	
Delirium is common in acute stroke patients	42 (89.3)
Delirium is an under diagnosed problem	44 (93.7)
Delirium is associated with high patient mortality	35 (74.5)
Delirium in stroke patients is rarely agitation	22 (46.8)
Delirium is challenging to assess in stroke patient	42 (89.4)
Current practices on delirium	
Question: In your opinion, what is the appropriate consideration and practice in handling delirium in stroke patients?	
Early detection through a standardised screening tool on high-risk patients with early management for delirium (Management)	44 (93.6)
Availability of adequate health personnel in managing stroke patients in order to avoid underdiagnosed delirium patient in acute stroke patients (Management)	28 (59.6)
Delirium care should be included as one of the basic training curriculum for the health personnel to familiarise and enhance knowledge (Management)	33 (70.2)
Proper structured evaluation procedure according to hospital policy on practice of delirium (Practice)	29 (61.7)
Attitudes on delirium	
Delirium is a normal part of acute stroke patient’s hospitalisation	22 (46.8)
Delirium should be monitored routinely	43 (91.5)
Delirium screening is a waste of time	0 (0.0)
Screening tools are complicated	8 (17.0)
Screening barriers on delirium	
Insufficient knowledge and training of delirium in acute stroke patients	38 (80.1)
Heavy workload caused lack of communication with patients and family	27 (57.4)
Lack of appropriate assessment tools	25 (53.2)
Lack of a structured evaluation procedure	31 (65.6)
Insufficient cooperation between physicians and allied health professionals	19 (40.4)
Time consumed for applying delirium screening tools	16 (34.0)
Health service organisation on delirium	
Do you know any guidelines in HSAAS for delirium risk identification, prevention, and management?	14 (29.8)
Are the equipment and devices sufficient? (Such as low-rise beds, call bells and clocks)	27 (57.4)

Notes:

* Percentage of Yes = %YES answers or % of the sum of ‘to some extend’, ‘agree’ and ‘strongly agree’ answers (from the 5-point Likert scale statements)

## Appendix

### SECTION A: SOCIO-DEMOGRAPHIC BACKGROUND

This section will identify your age, ethnicity, and gender. The results of this study will remain anonymous, and no respondent identification will be revealed.

1. Age
  □ 30 and below
  □ 31–40
  □ 41–50
  □ 50 and above
2. Ethnicity
  □ Malay
  □ Chinese
  □ Indian
  □ Others: Please state
3. Gender
  □ Female
  □ Male
4. Department in HPUPM
  □ Anaesthesiology and Critical care Department
  □ Regional Emergency Stroke Quick Response (RESQ) Department
  □ Dietetics Department
  □ Medical and Neurology Department
  □ Otorhinolaryngology, Head and Neck Surgery (ENT) Department (only speech therapist)
  □ Psychiatry Department
  □ Rehabilitation Medicine Department
  □ Radiology Department
5. Please choose your job title/ position.
  □ Nurse
  □ Assistant medical officer
  □ Dietician
  □ Speech Therapist
  □ Physiotherapist
  □ Occupational therapist
  □ Medical Officer
  □ Doctor-Specialistst
  □ Doctor – Consultant
6. Clinical experience
  □ less than 10 years
  □ 11 to 20 years
  □ 21 to 30 years
  □ 31 years or above
7. Knowledge/Educational level
  □ Diploma and below
  □ Degree
  □ Postgraduate diploma / Master
  □ Ph.D and above

### SECTION B: KNOWLEDGE OF DELIRIUM

This section will access your knowledge of delirium. Please choose only one best answer.

1. Have you received any training in delirium?
  □ Yes
  □ No
2. Among risk factors of delirium include a history of dementia, older age, and stroke.
  □ Yes
  □ No
3. I know how and when to do screening for delirium.
  □ Yes
  □ No
4. There is low prevalence of delirium among stroke patients in acute stroke units.
  □ Yes
  □ No

### SECTION C: CURRENT PRACTICES

This section will assess your current practices in managing stroke patients with delirium. You are required to choose answer(s) based on your opinion and experience. You are allowed to choose more than 1 answer.

1. In your opinion, what is the appropriate consideration and practice in handling delirium in stroke patients?
  □ Early detection through a standardised screening tool on high-risk patients with early management for delirium (management)
  □ Availability of adequate health personnel in managing stroke patients in order to avoid underdiagnosed delirium patients in acute stroke patients (management)
  □ Delirium care should be included as one of the basic training curricula for the health personnel to familiarise and enhance knowledge. (management)
  □ Proper structured evaluation procedure according to hospital policy on the practice of delirium (Practice)

### SECTION D: PERCEPTION

This section will assess your perceptions of delirium. Please choose only one best answer. It will be a scale from 1 (don’t know), 2 (don’t agree), 3 (to some extent), 4 (agree) or 5 (strongly agree).

1. Perception on delirium
  - Delirium is common in acute stroke patients.
    □ 1
    □ 2
    □ 3
    □ 4
    □ 5
  - Delirium is an under diagnosed problem
    □ 1
    □ 2
    □ 3
    □ 4
    □ 5
  - Delirium is associated with high patient mortality
    □ 1
    □ 2
    □ 3
    □ 4
    □ 5
  - Delirium in stroke patients are rarely agitated
    □ 1
    □ 2
    □ 3
    □ 4
    □ 5
  - Delirium is challenging to assess in stroke patient
    □ 1
    □ 2
    □ 3
    □ 4
    □ 5

### SECTION E: ATTITUDES

This section assesses your attitude towards delirium. Please choose only one best answer.

1. - Delirium is a normal part of acute stroke patients hospitalisation
    □ Yes
    □ No
    □ Not sure
  - Delirium should be monitored routinely.
    □ Yes
    □ No
    □ Not sure
  - Delirium screening is a waste of time.
    □ Yes
    □ No
    □ Not sure
  - Screening tools are complicated.
    □ Yes
    □ No
    □ Not sure

### SECTION F : SCREENING BARRIERS

This section will assess the screening barriers toward delirium identification. Please choose only one best answer.

1. - A. Insufficient knowledge and training of delirium in acute stroke patients
    □ Yes
    □ No
    □ Not sure
  - Heavy workload caused lack of communication with patients and family
    □ Yes
    □ No
    □ Not sure
  - Lack of appropriate assessment tools
    □ Yes
    □ No
    □ Not sure
  - Lack of a structured evaluation procedure
    □ Yes
    □ No
    □ Not sure
  - Insufficient cooperation between physicians and allied health professionals
    □ Yes
    □ No
    □ Not sure
  - Time consumed for applying delirium screening tools
    □ Yes
    □ No
    □ Not sure

### SECTION G: HEALTH SERVICE ORGANISATION

This section will assess your organisation policy and guidelines on delirium. In terms of the health service organisation, you are currently working in, please choose one relevant answer.

1. - Do you know of any guidelines in HPUPM for delirium risk identification, prevention and management?
    □ Yes
    □ No
  - Are the equipment and devices sufficient? (Such as low-rise beds, call bells and clocks)
    □ Yes
    □ No
